# Search for marker proteins to assess blood–brain barrier development

**DOI:** 10.3389/fnana.2026.1717532

**Published:** 2026-02-24

**Authors:** Yukari Shigemoto-Mogami, Kimiko Nakayama-Kitamura, Kaoru Sato

**Affiliations:** Laboratory of Neuropharmacology, Division of Pharmacology, National Institute of Health Sciences, Kawasaki, Kanagawa, Japan

**Keywords:** blood–brain barrier (BBB), development, markers, microphysiological system (MPS), permeability

## Abstract

The blood–brain barrier (BBB) separates the central nervous system from the peripheral blood circulation, and performs various functions such as dictating central nervous system (CNS)-specific pharmacokinetics, and maintaining brain homeostasis. While previous studies have clarified the mechanisms underlying cerebrovascular network development, details regarding *in vivo* BBB maturation remain unknown. In this regard, we previously reported that the development of physical tightness of the BBB and the formation of perivascular glial structures begins on postnatal day 4 and is almost complete by postnatal day 15. Moreover, the difficulty in predicting BBB permeability has hindered the development of CNS drugs, and microphysiological systems (MPSs) that mimic the BBB have been extensively developed to address this issue. Building on this information, in this study, we examined the temporal expression patterns of vascular proteins (CD31, Tie2, CD34, CD146, and agrin), tight junction proteins (ZO-1, claudin-5, and occludin), transporters (P-gp, BCRP, and Glut1), and transferrin receptors (TfRs) during the postnatal period, along with the development of BBB tightness. Based on their temporal expression patterns, these proteins were divided into five groups. We selected representative proteins from groups 1–5, respectively, and examined the temporal expression changes to determine the developmental stage of the BBB. The expression patterns of these proteins can be used to determine the maturation stage of BBB-MPSs.

## 1 Introduction

The blood–brain barrier (BBB) is a multicellular vascular complex that separates the central nervous system (CNS) from peripheral blood circulation, dictates CNS-specific pharmacokinetics, and maintains brain homeostasis (Abbott, 2013; Abbott et al., 2010; Hammarlund-Udenaes and Loryan, 2025; Pardridge, 2005). The BBB tightly controls the passage of molecules and ions, thereby protecting neurons from toxic substances, and delivers nutrients and oxygen to the brain (Keaney and Campbell, 2015; Obermeier et al., 2013; Zhao et al., 2015). The BBB is composed of brain vascular endothelial cells, the basement membrane, pericytes, and astrocyte endfeet. Vascular endothelial cells express tight junction (TJ) proteins that regulate physical tightness and transporters that modulate the orientation of permeable substances (Correale and Villa, 2009; Serlin et al., 2015; Sweeney et al., 2019).

Previous studies have clarified the mechanisms underlying the development of cerebrovascular networks (Coelho-Santos and Shih, 2020; Engelhardt and Liebner, 2014; Harb et al., 2013; Liebner et al., 2011; Wälchli et al., 2015), suggesting that BBB maturation begins rapidly after vascular network formation. However, *in vivo* BBB maturation remains understudied. In this regard, we previously reported that the development of physical tightness and perivascular glial structures starts on postnatal day (P) 4 and is almost complete by P15 in the rat brain cerebral cortex (Shigemoto-Mogami et al., 2024). Nevertheless, the difficulty in predicting BBB permeability has resulted a bottleneck in CNS drug development (Pardridge, 2005) for which microphysiological systems (MPSs) mimicking the BBB are being intensively developed (BBB-MPS). Accordingly, we previously proposed the minimum number of benchmark items required in the BBB-MPS for BBB-likeness (Nakayama-Kitamura et al., 2023). In this study, we aimed to investigate BBB maturation by examining the temporal expression patterns of vascular proteins, TJ proteins, transporters, and receptors during the postnatal development of physical tightness and perivascular glial structures. The findings obtained in this study have potential applications in the determination of the maturation stage of BBB-MPS and the qualification of the “Context of Use” (COU), the contextual needs leading to the specific goal.

## 2 Materials and methods

### 2.1 Animals and treatment

All animals used in this study were handled in accordance with the Guidelines for the Care and Use of Laboratory Animals published by the National Institute of Health Sciences. All experiments were approved by the Animal Research Committee of the National Institute of Health Sciences and conformed to relevant regulatory standards (approval number: 924). Wistar rats were purchased from Japan SLC (Shizuoka, Japan) and maintained under specific pathogen-free conditions at controlled temperature and humidity on a 12 h light/12 h dark cycle with ad libitum access to food and water. Ten to twelve rat pups were obtained from each pregnant rat litter that was subjected to dye penetration tests and immunostaining experiments at each postnatal time point, and inter litter comparisons were made. Three to six animals were used at all the time points in each experiment.

### 2.2 Determination of the timing of BBB formation in postnatal rats via tissue-fixable biotin administration

An experiment was conducted to evaluate the leakage of tissue-fixed biotin (sulfosuccinimidyl biotin; sulfo-NHS biotin; Thermo Fisher Scientific, Waltham, MA, USA) as previously described to evaluate the timing of BBB formation (Daneman et al., 2010; Shigemoto-Mogami et al., 2024). Male rats were transcardially perfused once with sulfo-NHS biotin (1 mg/mL) for 3–5 min at P1, 4, 7, 10, 15, and 30, followed by perfusion for 5 min with 4% paraformaldehyde (PFA) at a rate of 0.7 mL/min. Rat brains were then dissected and fixed in 4% PFA before being submerged in 30% sucrose. Subsequently, 30 μm thick sagittal sections of the embedded brains were cut, incubated at 24 °C for 3 h in a blocking solution (3% normal goat serum and 0.3% Triton X-100 in phosphate-buffered saline [PBS]), followed by incubation for 1 h at room temperature in a solution containing Alexa 546 streptavidin (1:500; S11225, Invitrogen, Waltham, MA, USA). After rinsing, the sections were stained with lectin (1:200; DL1177; Vector Labs, Newark, CA, USA) and 4′,6-diamidino-2-phenylindole (DAPI; 1:1000; 342-07431, Dojindo, Kumamoto, Japan). The stained sections were analyzed using a Nikon A1R confocal microscope system (Nikon, Tokyo, Japan) and quantified using NIS-Element analysis software (Nikon). Images with a ×20 field of view were obtained and eight blood vessels per image were measured. A line was drawn perpendicular to the blood vessel to quantify the fluorescence value and the highest biotin value on the vessel was set at 100. The sum of the fluorescence values on the line 20 μm left and right of the blood vessel was calculated and normalized to the highest value on the blood vessel. The fluorescence values of the lines in areas without blood vessels were used as the background and subtracted from the analysis data of the same image. At each time point, 112–128 blood vessel cross-sections in 14–16 field images of the cerebral cortex regions obtained from the three rats were quantitatively analyzed.

### 2.3 Immunohistochemical analysis of developmental changes in the configuration of BBB-composing cells and vascular expression of BBB functional proteins

The rats (P1, P4, P7, P10, P15, and P30) were anesthetized and perfused with PBS, followed by 4% PFA, and their brains were removed. Sagittal sections (30 μm thick) were sectioned from each half of the brain and incubated for 3 h at room temperature in the aforementioned blocking solution. Subsequently, the sections were incubated at 4 °C for 24 h with appropriate primary antibodies. The sections were then rinsed and incubated for 3 h at room temperature with corresponding secondary antibodies. Thereafter, the sections were rinsed and incubated for 1 h at room temperature in a solution containing lectin (1:200; DL1177; Vector Labs) and DAPI (1:1000; 342–07431; Dojindo). After a final round of rinsing, drying, and embedding in VectaShield (H-1000; Vector Labs), the stained sections were analyzed using a Nikon A1R-A1 confocal microscope. Images were obtained at intervals of 1.0 μm and 30 μm intervals in the z-direction and captured at a resolution of 1024 × 1024 pixels using a 60× field of view.

The following primary and secondary antibodies were used to assess developmental changes in the configuration of BBB-composing cells: guinea pig anti-ionized calcium binding adapter protein 1 (Iba1; 1:500; 019-9741; Fujifilm Wako Chemicals, Osaka, Japan) and rabbit anti-aquaporin-4 (AQP4; 1:500; A5971; Sigma-Aldrich, Darmstadt, Germany); anti-rabbit IgG-conjugated Alexa Fluorochrome or anti-guinea pig IgG-conjugated Alexa fluorochrome [1:1000; Invitrogen].

The following primary and secondary antibodies were used to assess developmental changes in the vascular expression of BBB functional proteins: mouse anti-CD31 (1:100; ab64543, Abcam; Cambridge, UK), rabbit anti-CD34 (1:250; ab81289, Abcam), rabbit anti-CD146 (1:250; ab75769, Abcam), mouse anti-Tie2 (1:250; ab24859, Abcam), rabbit anti-agrin (1:400; ab236652, Abcam), mouse anti-occludin (1:200; OC-3F10, Invitrogen), mouse anti-ZO-1 (1:100; 339100, Invitrogen), mouse anti-claudin-5 (1:200; 352500, Invitrogen), mouse anti-P-gp (1:50; GTX23364, Gene Tex; Irvine, CA, USA), rabbit anti-BCRP (1:1000; ab207732, Abcam), rabbit anti-Glut1 antibody (1:250; ab115730, Abcam), and mouse anti-Transferrin receptor (TfR) (1:250; 13-6800, Thermo Fisher Scientific); anti-rabbit, anti-mouse, or goat IgG-conjugated Alexa fluorochrome [Invitrogen; 1:1000].

### 2.4 Analysis of changes in the expression of BBB functional proteins in the capillaries

All images were acquired under the same imaging conditions (laser power and scan speed) for each parameter, and all signal intensities were calibrated using parenchyma without cells outside the blood vessels. Approximately 9–15 confocal images of the cerebral cortical regions were obtained from three male rat pups derived from three different litters at each age. Images of the blood vessels (lectin, red), nuclei (DAPI, blue), and candidate marker proteins (green) were analyzed using NIS-Elements AR. The lectin- and Iba1-positive microglial signals were removed using a cutting tool to extract only blood vessel signals. Additionally, the co-localized signals between the markers and blood vessels were detected, and the area of co-localized signals was normalized for total blood vessel area in each image to quantify the “marker-positive areas (M+)”.

### 2.5 Statistics

All fluorescence data are shown as averaged value ± SEM. Data were analyzed using an analysis of variance (ANOVA) followed by a Tukey’s multiple range test. Statistical significance was set as follows: *p < 0.05, **p < 0.01. vs. P1 or P4 value, while #*p* < 0.05, ##*p* < 0.01 vs. P10 or P15 value.

## 3 Results

### 3.1 Correlation of temporal expression of brain microvascular endothelial cell (BMEC) proteins with BBB integrity in the postnatal rat cerebral cortex

**Figure 1:**
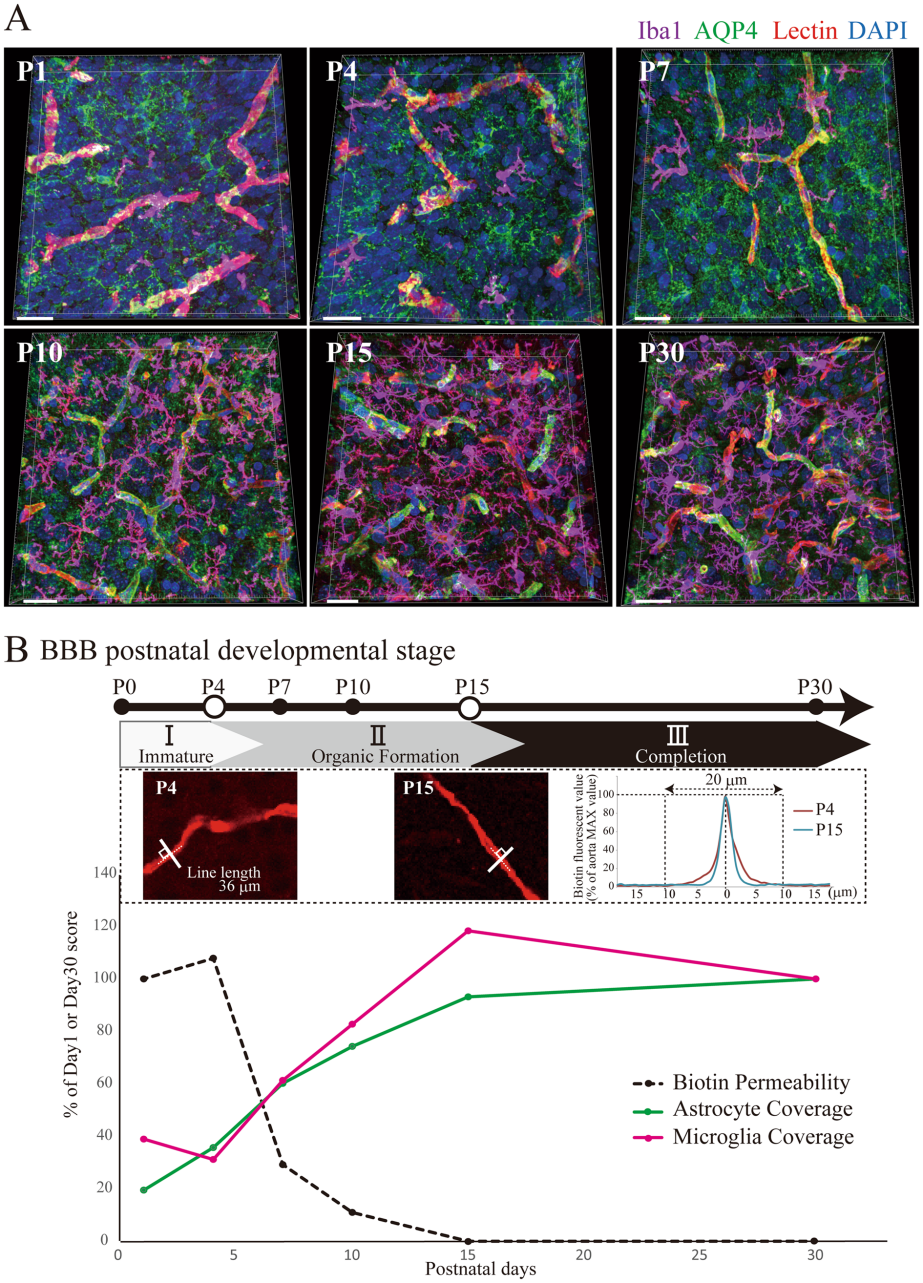
BBB maturation stages in the postnatal cerebral cortex based on glial configuration. **(A)** 3D images of AQP4, Iba1, lectin, and DAPI staining in the rat cerebral cortex at P1–30 are shown. Scale bar indicates 30 μm. AQP4 gradually accumulated in the blood vessels and localized to the blood vessels after P15. The number of Iba1+ microglia significantly increased after P10 and peaked at P15, changing their shape from ameboid to ramified. **(B)** Temporal changes in biotin permeability are corrected to a maximum value of 100%. Typical images of P4 and P15 rat brain cortical regions stained with streptavidin are shown. A solid line was drawn perpendicular to the blood vessel to quantify the fluorescence, which is indicated by the dashed line, and the red fluorescence value was measured, as shown in the upper right graph. The traces show the average of 70–76 transverse vessel lines for the respective postnatal ages. The maximum fluorescence value was set at 100, and the sum of the fluorescence values 20 μm to the left and to the right was calculated. The peak became sharper as the age increased. Astrocyte and microglial coverage rates were corrected to a P30 value of 100%. BBB formation and maturation in rats are classified into three phases based on biotin leakage and astrocyte and microglial contact with blood vessels (Shigemoto-Mogami et al., 2024). The phases are as follows: the “immature stage” when biotin permeability is still high; the “organic formation stage” when glial structures around blood vessels are formed; and the “completion stage” when the structures around blood vessels become stable and the morphology of microglia changes to a ramified type. Abbreviations: AQP4: aquaporin 4; BBB: blood–brain barrier; DAPI: 4′,6-diamidino-2-phenylindole; Iba1: ionized calcium binding adapter protein 1; P: postnatal day.

We verified our previous report on the development of the BBB and perivascular glial structures in the rat postnatal cerebral cortex until P30 (Figure 1). Figure 1A shows typical images of blood vessels (lectin+) and the surrounding glial cells (AQP4+ astrocytes and Iba1+ microglia). Figure 1B shows the time course of BBB permeability, indicated by the leakage of sulfo-NHS-biotin along perivascular glial structures. We quantified the physical tightness by measuring the leakage of sulfo-NHS-biotin, as shown in the upper panels of Figure 1B. The biotin signal values on the white line drawn perpendicular to the blood vessels were analyzed. The P15 biotin signal trace was sharper than the P4 trace, indicating that the leakage of sulfo-NHS-biotin was reduced over time (for more details, please see Section 2.2. in the Materials and Methods) from P4 onwards. Additionally, the values were almost the same at P15 and P30, indicating that BBB integrity plateaued at P15. Astrocytes covered most of the blood vessels from P15 onward (90.16 ± 2.94%), whereas microglia started to attach to the blood vessels by P4, rapidly increased after P7, with their morphologies changing from the amoeboid type to ramified type by approximately P15. The number and area of the microglia-blood vessel contacts peaked on P15 and decreased to 83.8 ± 5.6% of the maximum on P30. These data are consistent with our previous report, which showed that BBB physical development after birth can be divided into three stages based on BBB integrity and perivascular glial cell–blood vessel adhesion: immature (P0–P4), organizing (P4–P15), and complete (P15–P30) (Shigemoto-Mogami et al., 2024).

**Figure 2:**
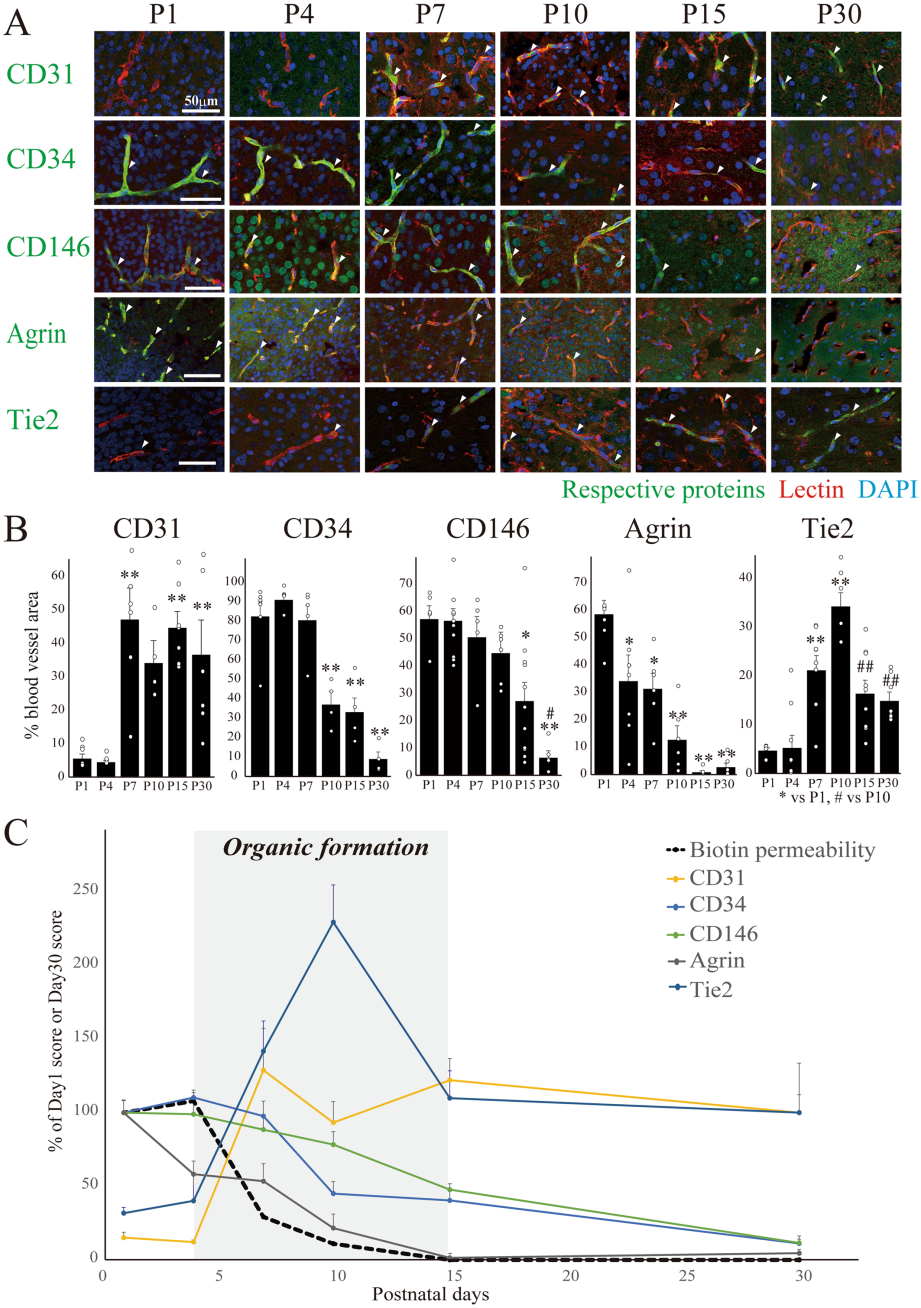
Expression of blood vessel developmental markers in the postnatal cerebral cortex. **(A)** Images of CD31, CD34, CD146, agrin, and Tie2 co-stained with lectin and DAPI in the rat cerebral cortex at P1–30 are shown. Scale bar indicates 50 μm. CD31 and Tie2 signals gradually increased in blood vessels, whereas CD34, CD146, and agrin signals gradually decreased. **(B)** The graphs show temporal changes in the percentage of marker+ area relative to the total vessel area. The colocalized signals between the markers and blood vessels were calculated, and the areas of the colocalized signals were normalized to the total blood vessel area in each image. Approximately 9–15 Images were obtained from the cerebral cortical regions of three rat pups of each age with a Nikon A1R-A1 confocal microscope. Data are shown as averaged value ± SEM. Data were analyzed using an ANOVA followed by a Tukey’s multiple range test. *p < 0.05, **p < 0.01. vs. P1 value, while #*p* < 0.05, ##*p* < 0.01 vs. P10 value. **(C)** Graph summarizing results for B and Figure 1 B. Changes in biotin permeability, CD34, CD146, and agrin were corrected to a P1 value of 100%, and the astrocyte coverage rate, microglial coverage rate, CD31, and Tie2 were corrected to a P30 value of 100%. The grey zone represents the organic formation term (P4P15). Abbreviations: CD31, cluster of differentiation 31 or platelet endothelial cell adhesion molecule 1; CD146, melanoma cell adhesion molecule; DAPI: 4′,6-diamidino-2-phenylindole; P, postnatal day; Tie2, TEK receptor tyrosine kinase; ANOVA, analysis of variance.

Subsequently, we examined the temporal expression of postnatal vascular proteins until P30. We evaluated the expression of CD31, CD34, CD146, agrin, and Tie2 (Figure 2), which are established markers of angiogenesis and vascular differentiation. As shown in Figures 2A,B, the CD31+ area in the blood vessels rapidly increased at P7 and plateaued thereafter. In contrast, the CD34+ area decreased with a peak at P4. The CD146+ area gradually decreased from P1. Notably, CD146 was also observed in non-vascular areas up to P7. Most blood vessel areas at P1 were agrin+, which decreased rapidly after birth and became undetectable after P15. The Tie2+ area increased until P10 and decreased thereafter. The time courses of the areas positive for the respective proteins are shown as percentages of the blood vessel area in Figure 2B. Each protein exhibited a unique temporal expression pattern until P30.

### 3.2 Expression of TJ proteins and BBB functional proteins on P4, P15, and P30

**Figure 3:**
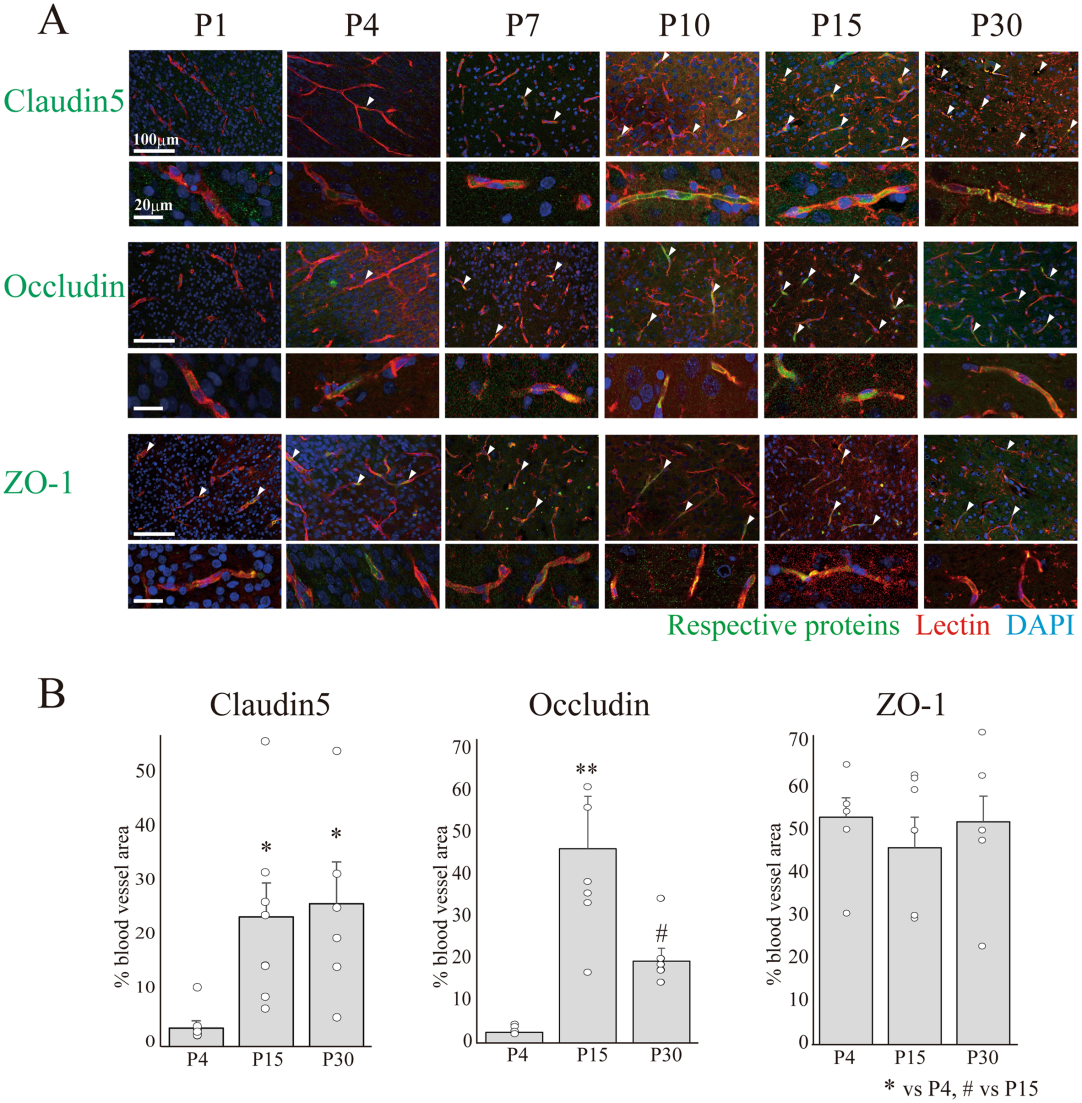
Analysis of vascular TJ protein expression in the postnatal cerebral cortex. **(A)** Images of claudin-5, occludin, and ZO-1 co-stained with lectin and DAPI in the rat cerebral cortex at P1–30. Scale bar indicates 100 μm. Claudin-5 and occludin signals increased in blood vessels during development, whereas ZO-1 signals remained unchanged. **(B)** The graphs show changes in the percentage of marker+ area of the total vessel area at P4, P15, and P30. The colocalized signals between the markers and blood vessels were calculated, and the areas of the colocalized signals were normalized to the total blood vessel area in each image. Approximately 9–15 images were obtained from the cerebral cortical regions of three rat pups from each age group using a Nikon A1R-A1 confocal microscope. Data are shown as averaged value ± SEM. Data were analyzed using an ANOVA followed by a Tukey’s multiple range test. *p < 0.05, **p < 0.01 vs. P4 value, and #*p* < 0.05 vs. P15 value. DAPI: 4′,6-diamidino-2-phenylindole; P, postnatal day; TJ, tight junction; ZO-1: zonula occludens-1; ANOVA, analysis of variance.

**Figure 4:**
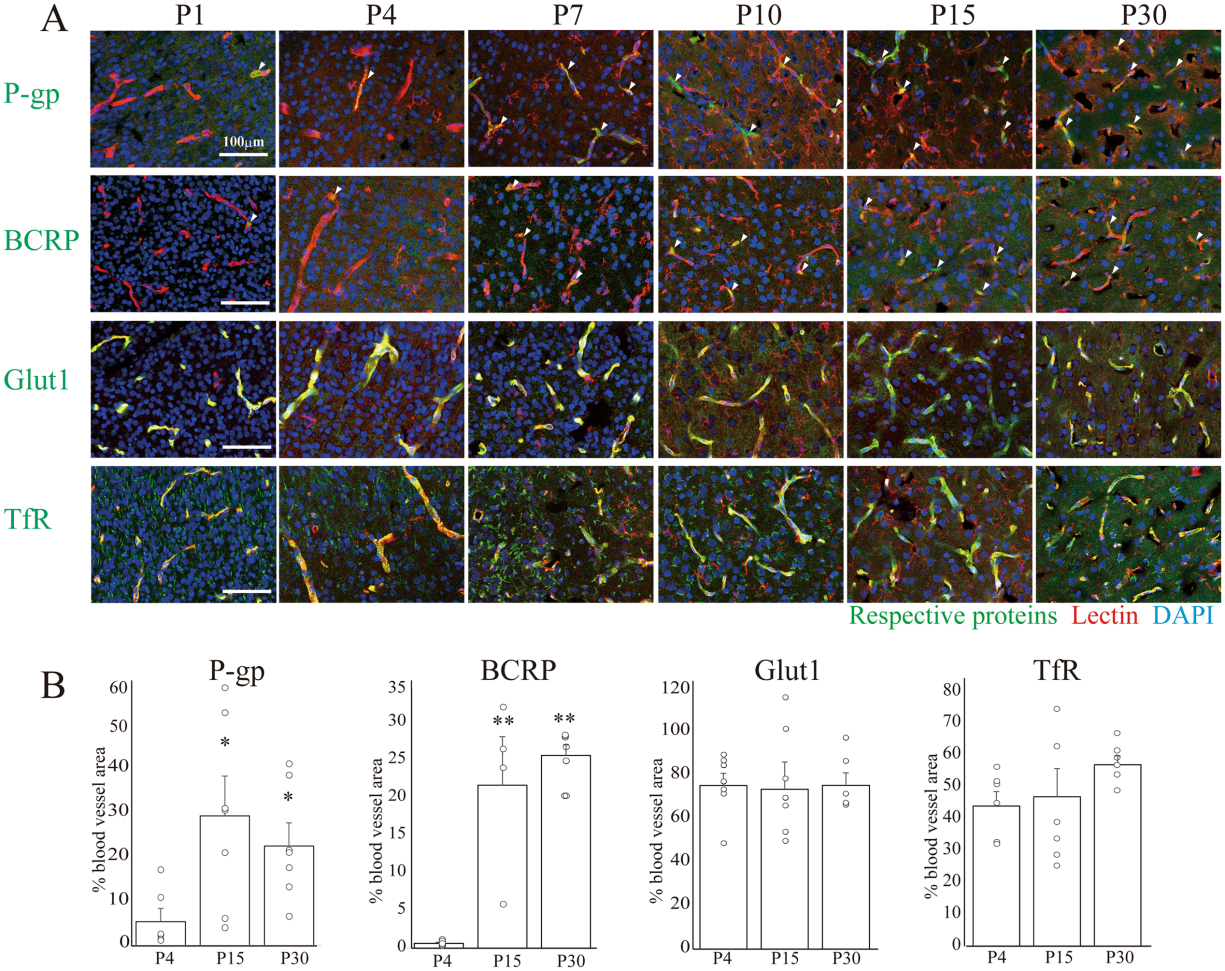
Analysis of vascular expression of transporters and receptor proteins in the postnatal cerebral cortex. **(A)** Images of P-gp, BCRP, Glut1, and TfR co-stained with lectin and DAPI in the rat cerebral cortex at P1–30. Scale bar indicates 100 μm. The arrowhead shows the co-localization areas of the marker and lectin. P-gp and BCRP signals gradually increased in the blood vessels during development, whereas Glut1 and TfR signals were high in the blood vessels at P1 and remained unchanged. **(B)** The graphs show changes in the percentage of marker+ area of the total vessel area at P4, P15, and P30. The colocalized signals between the markers and blood vessels were calculated, and the areas of the colocalized signals were normalized to the total blood vessel area in each image. Approximately 9–15 images were obtained from the cerebral cortical regions of three rat pups from each age group using a Nikon A1R-A1 confocal microscope. Data are shown as averaged value ± SEM. Data were analyzed using an ANOVA followed by a Tukey’s multiple range test. *p < 0.05, **p < 0.01 vs. P4 value. Abbreviations: BCRP: breast cancer resistance protein; DAPI: 4′,6-diamidino-2-phenylindole; Glut1: glucose transporter type 1; P: postnatal day; P-gp: P-glycoprotein; TfR: transferrin receptor; ANOVA, analysis of variance.

As previously reported by our team, the minimal essential benchmarks for BBB-likeness (Nakayama-Kitamura et al., 2023) include the expression of TJ proteins, transporters, and TfR. Therefore, we examined the expression of these proteins in vascular endothelial cells at P4, P15, and P30 (Figures 3, 4). Among the TJ proteins, we examined changes in the areas positive for the anchor proteins claudin-5, occludin, and ZO-1, which are important for maintaining a strong barrier function (Alahmari, 2021; Greene and Campbell, 2016) (Figure 3). The claudin-5+ and occludin+ areas increased after birth until P15 and thereafter remained constant until P30. Conversely, the ZO-1+ area was approximately 50% at P4 and remained constant until P30. We then examined the expression of P-gp, BCRP, Glut1, and TfR, key molecules closely related to the pharmacokinetics (PK) and pharmacodynamics (PD) in the CNS (Banks et al., 2024; Löscher and Potschka, 2005). Figure 4A shows the temporal expression of P-gp, BCRP, Glut1, and TfR from P1 to P30. As shown in Figure 4B, the P-gp + and BCRP+ areas in the blood vessels increased until P15, and decreased slightly by P30, but remained constant. Conversely, Glut1 was widely expressed on P4 (75.2 ± 5.6%) and the Glut1 + area in the blood vessels remained constant until P30. Meanwhile, the TfR + area occupied 44.1 ± 4.5% of blood vessels, and this ratio remained unchanged until P30.

### 3.3 Summary of the developmental changes in the areas positive for the vascular BMEC proteins

**Figure 5:**
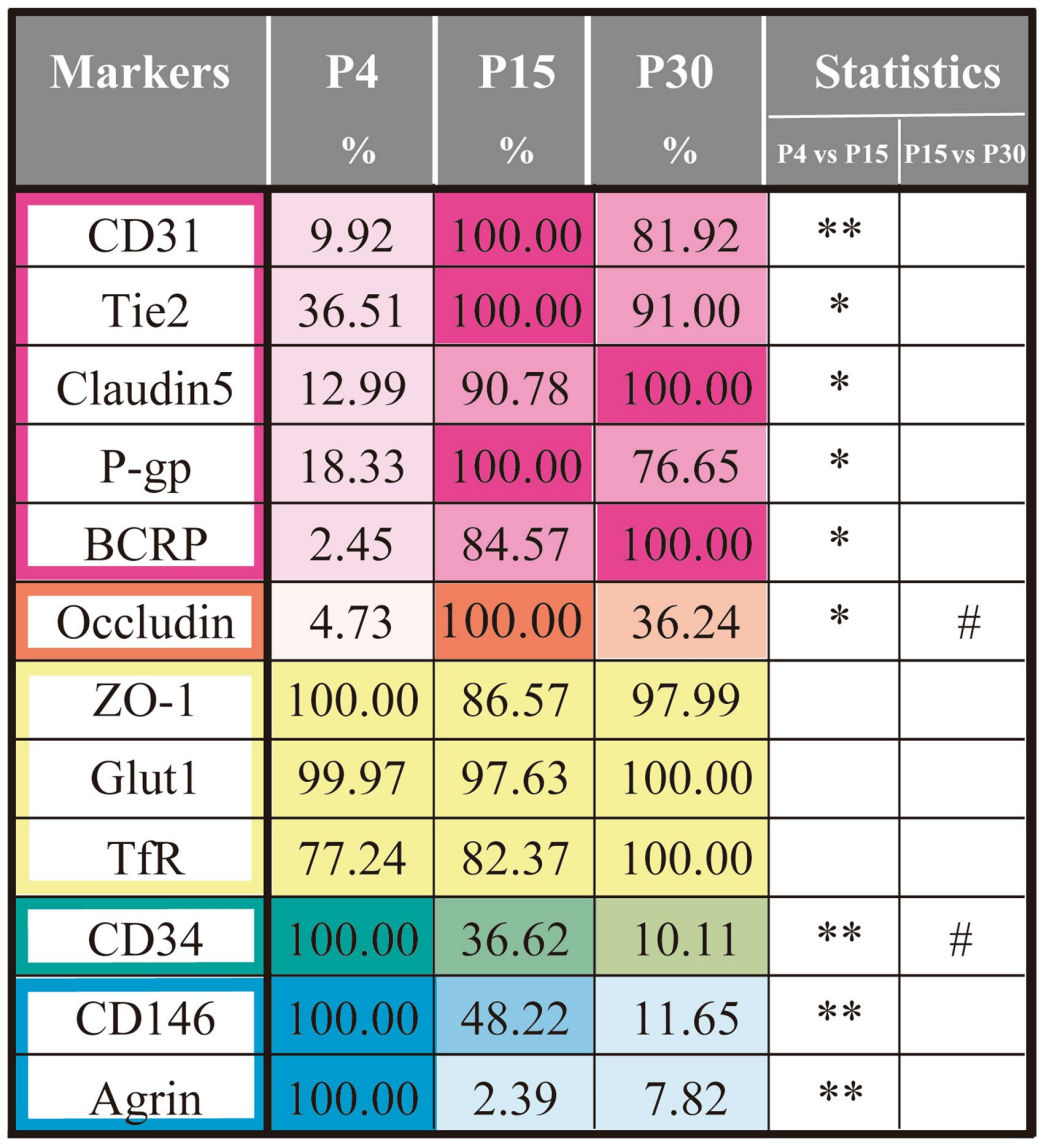
Summary of the developmental changes in the areas positive for the vascular BMEC proteins. The area values of BBB marker proteins expressed in the blood vessels of the cerebral cortex on P4, P15, and P30 are shown. Pink indicates increased BBB proteins from P4 to P15; orange indicates occluding with transient increase at P15; green indicates significant decrease at P15 and P30; blue indicates decreased BBB proteins at P15. Data are shown as averaged value ± SEM. Data were analyzed using an ANOVA followed by Tukey’s multiple tests. *p < 0.05, **p < 0.01 vs. P4 value, while #*p* < 0.05 vs. P15 value. Abbreviations: BBB, blood–brain barrier; Iba1, ionized calcium-binding adapter protein 1; P, postnatal day.

A comparison of the positive area ratios in the blood vessels at P4, P15, and P30 revealed that the vascular endothelial proteins evaluated in this study (CD31, CD34, CD146, agrin, Tie2, claudin-5, occludin, ZO-1, P-gp, BCRP, Glut1, and TfR) could be categorized into five groups based on their temporal expression patterns, as shown in Figure 5. Group 1 comprised the areas of CD31+, Tie2+, claudin-5+, P-gp+, and BCRP+ in the blood vessels, which increased significantly from P4 to P15, although the difference between P15 and P30 was not significant. Group 2 included the occludin+ area, which increased significantly from P4 to P15 and decreased significantly from P15 to P30. Group 3 comprised the Glut1+, TfR+, and ZO-1+ areas, which remained unchanged after birth. Group 4 included the CD34+ area, which decreased significantly from P4 to P15 and further decreased from P15 to P30. Finally, group 5 included the CD146+ and agrin+ areas, which decreased significantly from P4 to P15, whereas the difference between P15 and P30 was not significant. The expression patterns of these five protein groups are important for determining the developmental stages of the BBB.

## 4 Discussion

In this study, we investigated the *in vivo* temporal expression patterns of various BBB proteins from the postnatal period until P30 in a rat model. P30 rats are adolescents used to investigate behavior, learning, and memory, indicating that the processes necessary for brain architecture are completed by P30 rats. Although previous studies have clarified the mechanisms underlying the development of cerebrovascular networks (Coelho-Santos and Shih, 2020; Engelhardt and Liebner, 2014; Harb et al., 2013; Liebner et al., 2011; Wälchli et al., 2015), little information is available regarding *in vivo* BBB maturation following the completion of angiogenesis from P14 to P21 (Engelhardt and Liebner, 2014; Liebner et al., 2011). In our previous study, we have clarified the interactive development of BBB tightness and perivascular glial cells (astrocytes and microglia) (Shigemoto-Mogami et al., 2024). We reported three stages based on the development of perivascular glial structures and BBB physical tightness (the immature stage [P0–P4], organizing stage [P4–P15], and complete stage [P15–P30]). We quantified BBB physical tightness by measuring the leakage of sulfo-NHS-biotin in previous and present studies. Transendothelial electrical resistance (TEER) and solute tracer flux have also been used for this purpose. However, both these methods are difficult to apply *in vivo*. The blood vessel area is necessary for TEER calculation, and it is necessary to select a suitable size/structure for solute tracer flux measurement.

We first examined the temporal expression of vascular markers, such as CD31, CD34, CD146, agrin, and Tie2. CD31 is located near the TJ complex and functions as a cell adhesion molecule in mature endothelial cells (Zhang et al., 2023). CD31 actively contributes to BBB integrity (Wimmer et al., 2019). Based on this, CD31 has become a representative marker of mature blood vessels. Our data showed that the CD31+ area in blood vessels increased remarkably at P7. This high expression level persisted from P7 onward. Since physical tightness reached the adult level at P15, it is suggested that the physical tightness become matured around P15 after CD31 expression reached a maximum level around P7. CD34 is a transmembrane phosphoglycoprotein that is expressed in vascular endothelial cell precursors in blood vessels that lack TJ proteins during angiogenesis (Sidney et al., 2014). Our results showed that the CD34 + area in the blood vessels dramatically decreased after P10 and continued to decrease until P30. CD146 is an adhesion molecule that regulates angiogenesis, vascular permeability, and leukocyte trafficking (Leroyer et al., 2019). Therefore, CD146 can be used as a marker for immature vascular endothelial cells. After BBB maturation, CD146 expression is limited to the pericytes (Chen et al., 2017). In this study, most areas of the blood vessels were CD146+ from P1 to P10, and the expression kept decreasing until P30. The temporal expression patterns of CD34 and CD146 raise the possibility that immature endothelial cells remain when the integrity get matures at P15 and gradually decrease until P30. Agrin, a heparan sulfate proteoglycan found in the extracellular matrix of basement membranes, regulates the distribution of AQP4 in astrocytic endfeet (Noell et al., 2009). We found that the agrin+ area decreased to a very low level after P15. Because astrocyte coverage visualized by the AQP4 signal gradually increased and reached the P30 level at P15, agrin may be important during the initial step of astrocyte endfeet formation. Tie2 is a receptor tyrosine kinase predominantly expressed in vascular endothelial cells (Sato et al., 1995). Tie2 has two ligands, angiopoietins 1 and 2 (Ang1 and Ang2), which have opposite effects; Ang1 strengthens cell–cell junctions and enhances endothelial cell survival, whereas Ang2 can antagonize these effects (Li et al., 2020). Tie2+ area increased in an age-dependent manner until P10, decreased at P15, and remained unchanged until P30. The balance between the two ligands and Tie2 might be related to the maturation of the physical tightness.

The expression of claudin-5, occludin, and ZO-1 was examined as representative TJ proteins. Claudin-5 is critical for the BBB integrity (Jia et al., 2014). The vascular claudin-5+ area increased remarkably at P15 and remained constant until P30, which coincided with the maturation pattern of physical tightness. Occludin levels transiently increased at P15. Occludin is the predominant regulator of TJ function (Overgaard et al., 2011). Recently, it was shown that the interaction between the long noncoding RNA SNHG12 and occludin blocks the ubiquitin-proteasome system, which is critical for the maintenance of BBB integrity (Li et al., 2022). The relationship between the transient increase at P15 and the above functions may be the next challenge. In contrast to transmembrane TJ proteins such as claudin5 and occludin, ZO-1 is a tight junction anchor protein expressed at an embryonic age (Nico et al., 1999). ZO-1 also functions as a cytoplasmic accessory protein (Zheng et al., 2023). ZO-1 organizes the proteins that interact with the actin cytoskeleton in the plasma membrane. This dual role of ZO-1 may be related to its stable expression throughout the study period.

P-gp and BCRP are ATP-binding cassette transporters that actively efflux drugs and other substances from the brain (Chai et al., 2022; Löscher and Potschka, 2005). These transporters affect bioavailability and are important for predicting PK and PD in the CNS. P-gp is an essential component of the detoxification system, managing endogenous neurotoxic substrates such as amyloid-*β* peptides, steroids, bile acids, and metabolites (Gil-Martins et al., 2020). Our data indicated that the expression of these efflux transporters peaked at P15, which is consistent with the completion of physical tightness maturation. Glut1 delivers L-glucose to the brain. The vascular Glut1+ area was >70% at P4 and remained almost constant until P30. Because L-glucose is the main energy source in the brain, stable expression during the entire period is reasonable. TfR delivers holotransferrin (iron-bound transferrin) and maintains iron homeostasis in the brain. As iron is essential for neuronal development, neurotransmitter synthesis, and myelination (Gao et al., 2025), a stable expression level is also reasonable.

Proteins were divided into five groups based on their temporal expression patterns (Figure 5). Group 1 included CD31, Tie2, claudin5, P-gp, and BCRP (pink columns), which showed a rapid increase at P15, and the level at P15 was almost the same as that at P30. Temporal expression of these proteins correlates with the maturation of the BBB. Group 2 contained occludin (orange columns), which rapidly increased at P15 and decreased at P30. Whether this transient increase is related to BBB maturation remains to be determined. Group 3 included ZO-1, Glut1, and TfR (yellow columns), which showed little change throughout the study period, suggesting that these proteins were essential for neural cell survival. The levels of group 4 (CD34) and group 5 (CD146 and agrin) proteins decreased with age, suggesting that these proteins have specific roles in the early developmental stages. To investigate the BBB maturation process more precisely, we will confirm the temporal changes in functional activities of the respective proteins with age in future studies.

This classification is important for the development of *in vitro* BBB models. In response to the Food and Drug Administration (FDA) Modernization Act 2.0/3.0, MPSs mimicking the BBB have been developed. The respective BBB-MPSs have unique designs including organoids, which are suitable for the “Context of Use” (COU) in drug development (Bicker et al., 2014; Chaulagain et al., 2023). However, no standard method has been established to determine the maturation stage of BBB-MPSs. At present, the maturation stage of BBB-MPS is estimated by a combination of TEER values, the expression of marker proteins, and the permeability of control compounds, whose *in vivo* permeability is already known. However, this information is insufficient to determine the extent to which the BBB-MPS reflects a mature BBB in vivo. This grouping approach can relate the BBB-MPS to the in vivo BBB by adopting the temporal protein expression pattern as a common parameter, both in vitro and in vivo. Examining the temporal expression patterns of representative proteins from groups 1–5 in vascular endothelial cells could be used to estimate the developmental stages of BBB-MPS relative to those in vivo. As the practical “starter set,” we recommend CD31 (group1), occludin (group2), Glut1 (group3), CD34 (group4), and CD146 (group5), the blood vessel markers (CD31, CD34, CD146) and the basic functional proteins (occludin and Glut1). For a more precise qualification of BBB-MPS for COU, we recommend that the functions of specific target proteins be individually evaluated. Currently, we are attempting to establish a BBB-MPS by installing human-induced pluripotent stem cell-derived BBB cells. A comparison of data obtained by these BBB-MPSs would be helpful for investigating species differences and improving human predictability.

## Data Availability

The original contributions presented in the study are included in the article/supplementary material, further inquiries can be directed to the corresponding author.
